# Nanoerythrosome-functionalized biohybrid microswimmers

**DOI:** 10.1063/1.5130670

**Published:** 2020-04-07

**Authors:** Nicole Buss, Oncay Yasa, Yunus Alapan, Mukrime Birgul Akolpoglu, Metin Sitti

**Affiliations:** 1 Physical Intelligence Department, Max Planck Institute for Intelligent Systems, 70569 Stuttgart, Germany; 2 Technical Biology, University of Stuttgart, 70174 Stuttgart, Germany; 3 School of Medicine and School of Engineering, Koç University, 34450 Istanbul, Turkey

## Abstract

Biohybrid microswimmers, which are realized through the integration of motile microscopic organisms with artificial cargo carriers, have a significant potential to revolutionize autonomous targeted cargo delivery applications in medicine. Nonetheless, there are many open challenges, such as motility performance and immunogenicity of the biological segment of the microswimmers, which should be overcome before their successful transition to the clinic. Here, we present the design and characterization of a biohybrid microswimmer, which is composed of a genetically engineered peritrichously flagellated *Escherichia coli* species integrated with red blood cell-derived nanoliposomes, also known as nanoerythrosomes. Initially, we demonstrated nanoerythrosome fabrication using the cell extrusion technique and characterization of their size and functional cell membrane proteins with dynamic light scattering and flow cytometry analyses, respectively. Then, we showed the construction of biohybrid microswimmers through the conjugation of streptavidin-modified bacteria with biotin-modified nanoerythrosomes by using non-covalent streptavidin interaction. Finally, we investigated the motility performance of the nanoerythrosome-functionalized biohybrid microswimmers and compared it with the free-swimming bacteria. The microswimmer design approach presented here could lead to the fabrication of personalized biohybrid microswimmers from patients' own cells with high fabrication efficiencies and motility performances.

## INTRODUCTION

Biohybrid microswimmers are mainly composed of integrated biological actuators and synthetic cargo carriers and have been recently shown to be promising toward minimally invasive theranostic applications.^1–4^ Various microorganisms, including bacteria,^5,6^ microalgae,^7,8^ and spermatozoids,^9,10^ have been utilized to fabricate different biohybrid microswimmers with advanced medical functionalities, such as autonomous control with environmental stimuli for targeting, navigation through narrow gaps, and accumulation to necrotic regions of tumor environments.^11^ Steerability of the synthetic cargo carriers with long-range applied external fields, such as acoustic or magnetic fields,^12,13^ and intrinsic taxis behaviors of the biological actuators toward various environmental stimuli, such as chemoattractants,^14^ pH, and oxygen,^15,16^ make biohybrid microswimmers a promising candidate for a broad range of medical active cargo delivery applications.^11^ However, translation of the biohybrid microswimmers from bench to bedside needs further investigation of ease of handling, payload efficiency, biocompatibility, biodegradability, non-immunogenicity, and penetration capabilities through complex biological environments including the mucosa and extracellular matrices.^17,18^

Bacteria have a high swimming speed and efficiency in the low Reynolds (Re) number flow regime, are capable of sensing and responding to external environmental signals, and could be externally detected via fluorescence or ultrasound imaging techniques.^19–21^ Due to their inherent sensing capabilities, various bacteria species have been investigated as potential anti-tumor agents and have been the subject of preclinical and clinical trials.^22–27^ Furthermore, the presence of different bacteria species in the human body, such as on the skin and the gut microenvironment, has promoted their use as potential theranostic agents or carriers in several medical applications.^28^ On the other hand, specialized eukaryotic cells, such as red blood cells (RBCs), are one of the nature's most efficient passive carriers with high payload efficiency, deformability, degradability, and biocompatibility, and have also been used in various medical applications.^29–31^ RBCs and RBC-derived nanovesicles, such as nanoerythrosomes, have been successfully adopted as passive cargo carriers to enhance the circulation time of the applied substances in the body,^32^ and to deliver different bioactive substances for the treatment of various diseases observed in the liver, spleen and lymph nodes, and also cancer via administrating through intravenous, intraperitoneal, subcutaneous, and inhalational routes.^33–37^ For instance, decreased recognition of drug-loaded particles by immune cells was shown when attached to membranes of the RBCs prior to intravenous injection into mice.^38^ Additionally, the altered bioaccumulation profile of nanocarriers was shown when conjugated onto the RBCs, boosting the delivery of nanocarriers to the target organs.^39^ It was also reported that the half-life of Fasudil, a drug for pulmonary arterial hypertension, inside the body increased approximately sixfold to eightfold when it was loaded into nanoerythrosomes.^37^

Superior cargo-carrying properties of the RBCs have also generated increased interest for their use in biohybrid microswimmer designs. Recently, active navigation and control of drug and superparamagnetic nanoparticle (SPION)-loaded RBCs were presented using sound waves and magnetic fields.^12,40^ RBCs were further utilized in the fabrication of soft biohybrid microswimmers powered by motile bacteria for active cargo delivery applications.^13^ RBCs, loaded with drug molecules and SPIONs, were propelled by bacteria and steered via magnetic fields, which were also capable of traveling through gaps smaller than their size due to the inherent high deformability of the RBCs. Despite advanced functionalities of the biohybrid microswimmers enabled by RBCs, there is still a great need for next-generation microswimmer designs with better propulsion performance in complex biological environments.

**FIG. 1.**
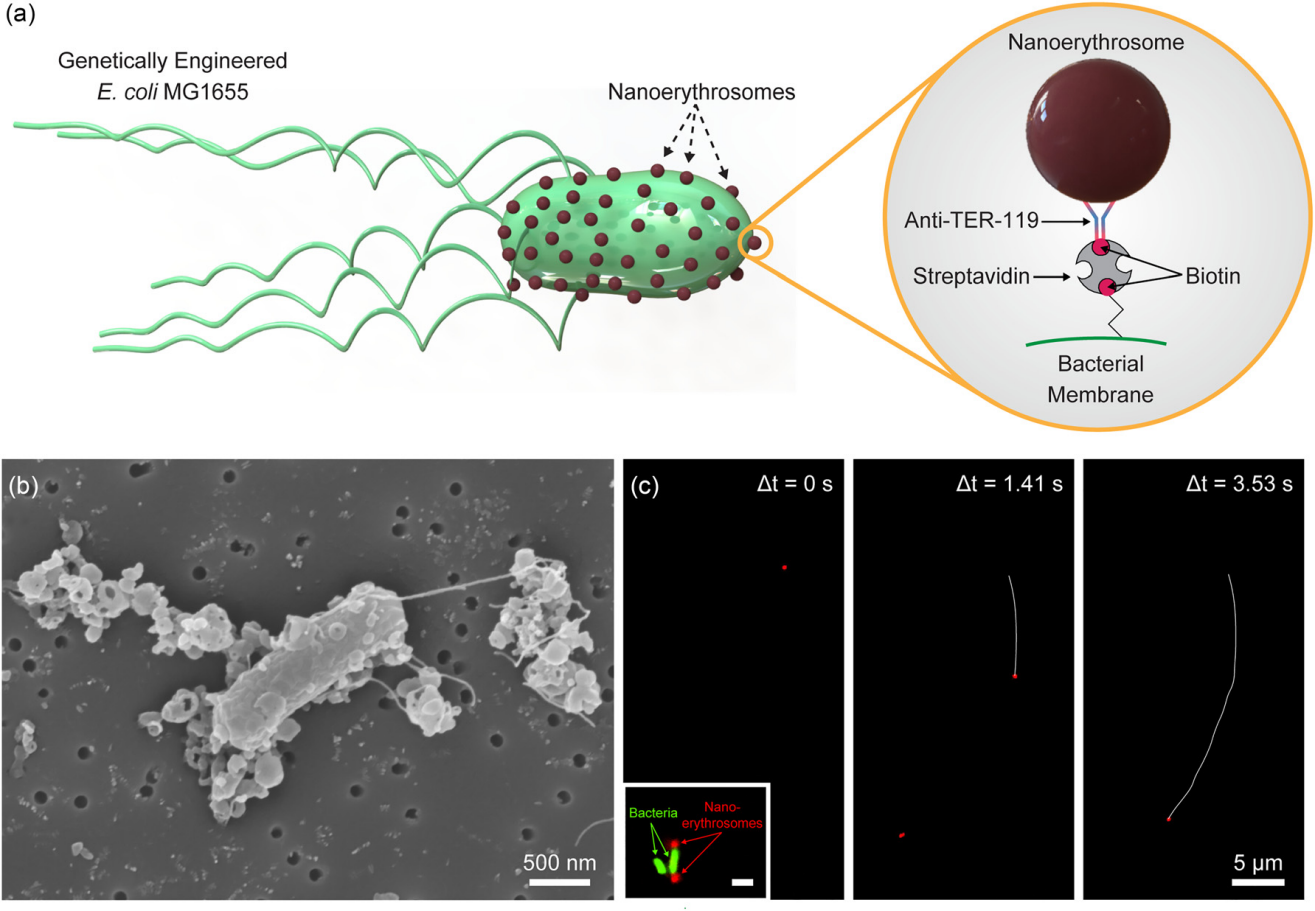
Biohybrid bacterial microswimmers functionalized with red blood cell (RBC)-membrane nanoliposomes (nanoerythrosomes). (a) Biohybrid bacterial microswimmers are composed of genetically engineered motile *E. coli* MG1655 substrain and nanoerythrosomes made out of the RBC membranes. Nanoerythrosomes are functionalized on the bacterial membrane via biotin–streptavidin–biotin interaction. The nanoerythrosome membrane is biotinylated by functionalization of TER-119 antibodies with biotin molecules, whereas *E. coli* MG1655 is bioengineered to express biotin attachment peptides on its membrane and biotin molecules are directly conjugated on its membrane surface. (b) Scanning electron microscopy (SEM) image of the nanoerythrosome functionalized bacterial swimmers. Nanoerythrosomes were conjugated not only to the bacterial body but also to their flagella. (c) A typical swimming trajectory for the biohybrid bacterial microswimmers captured via fluorescent microscopy. The red dot indicates nanoerythrosome on bacteria and the white line shows the swimming trajectory. The inset shows bacteria (green color) functionalized with nanoerythrosomes (red color). The scale bar of the inset is 1 *μ*m.

In this context, RBC-derived nanoerythrosomes stand out as strong candidates as carriers in biohybrid microswimmer designs. Nanoerythrosomes possess the very same membrane properties as the RBCs with small sizes that could enhance motility and penetration of biohybrid microswimmers. In this study, we present a biohybrid microswimmer design realized by the functionalization of a genetically engineered *Escherichia coli* MG1655 substrain with nanoerythrosomes [Fig. 1(a)]. RBCs were isolated from whole murine blood and nanoerythrosomes were fabricated from emptied RBCs, also known as RBC ghosts, using the cell extrusion technique as previously described with some modifications.^41^ Dynamic light scattering (DLS) and scanning electron microscopy (SEM) characterizations showed a size range of 100 nm–1 *μ*m nanoerythrosomes. Preservation of important RBC membrane proteins was confirmed by fluorescence microscopy and flow cytometry analyses. The functionalization of biotin-displaying bacteria with biotinylated nanoerythrosomes was realized by streptavidin coupling and showed via fluorescence microscopy. Finally, the motility performance of the fabricated biohybrid microswimmers in comparison to free bacteria was investigated using a two-dimensional (2D) object tracking algorithm. The biohybrid microswimmers showed enhanced swimming speeds compared to the previously reported biohybrid microswimmer designs powered by various *E. coli* strains. The biohybrid microswimmer fabrication approach presented here could enable the next generation of high-yield and high-performance autonomous biohybrid microswimmers with personalized nanocarriers from patients' own cells.

## RESULTS

Fabrication of the nanoerythrosome-functionalized biohybrid microswimmers was realized through the conjugation of streptavidin-modified genetically engineered *E. coli* MG1655 substrain with biotin-modified nanoerythrosomes [Fig. 1(a)]. The genetically engineered bacteria species contained a pOS233 plasmid which could be induced using isopropyl-β-D-thiogalactopyranoside (IPTG) to encode biotin acceptor peptides on the cell membrane of the bacteria through autotransporter antigen 43 [Fig. S1(a)].^42^ Such bacteria species also contained another plasmid that could be induced using *L*-arabinose for enhanced green fluorescent protein expression, which was used for fluorescence detection of the bacteria [Fig. S1(b)]. Initially, growth rates of this bacteria species were investigated from single colonies when they were induced with IPTG, *L*-arabinose or both IPTG and *L*-arabinose to determine the changes of the bacterial growth characteristics upon induction of the plasmids for the expression of the corresponding genes (Fig. S2). A decreased growth rate of the bacteria was observed when they were induced with both IPTG and *L*-arabinose simultaneously, which may be due to the increased metabolic burden. The experiments were performed according to the obtained bacterial growth characteristics. Briefly, the genetically engineered bacteria species were cultured overnight and then induced with IPTG the next day until their OD_600_ reached 0.6. After the induction of the proper genetic sequence of the plasmid DNA, the bacteria species had biotin molecules on their cell membranes. They were further incubated with streptavidin molecules for 1 h to modify the bacterial membrane for nanoerythrosome conjugation experiments. In parallel, nanoerythrosomes were modified with biotin molecules via biotin-conjugated anti-TER119 antibodies for 1 h to realize the non-invasive conjugation of the bacterial actuators with the cargo carriers [Fig. 1(a)]. TER119 proteins are considered as lineage markers due to their high expression levels on the mature erythrocyte membrane.^43^ The SEM analyses showed the proper conjugation of the nanoerythrosomes with the streptavidin-modified genetically engineered bacteria species [Fig. 1(b)]. The fabricated biohybrid microswimmers powered and carried the nanoerythrosomes as demonstrated with the fluorescence microscopy images [Fig. 1(c)].

**FIG. 2.**
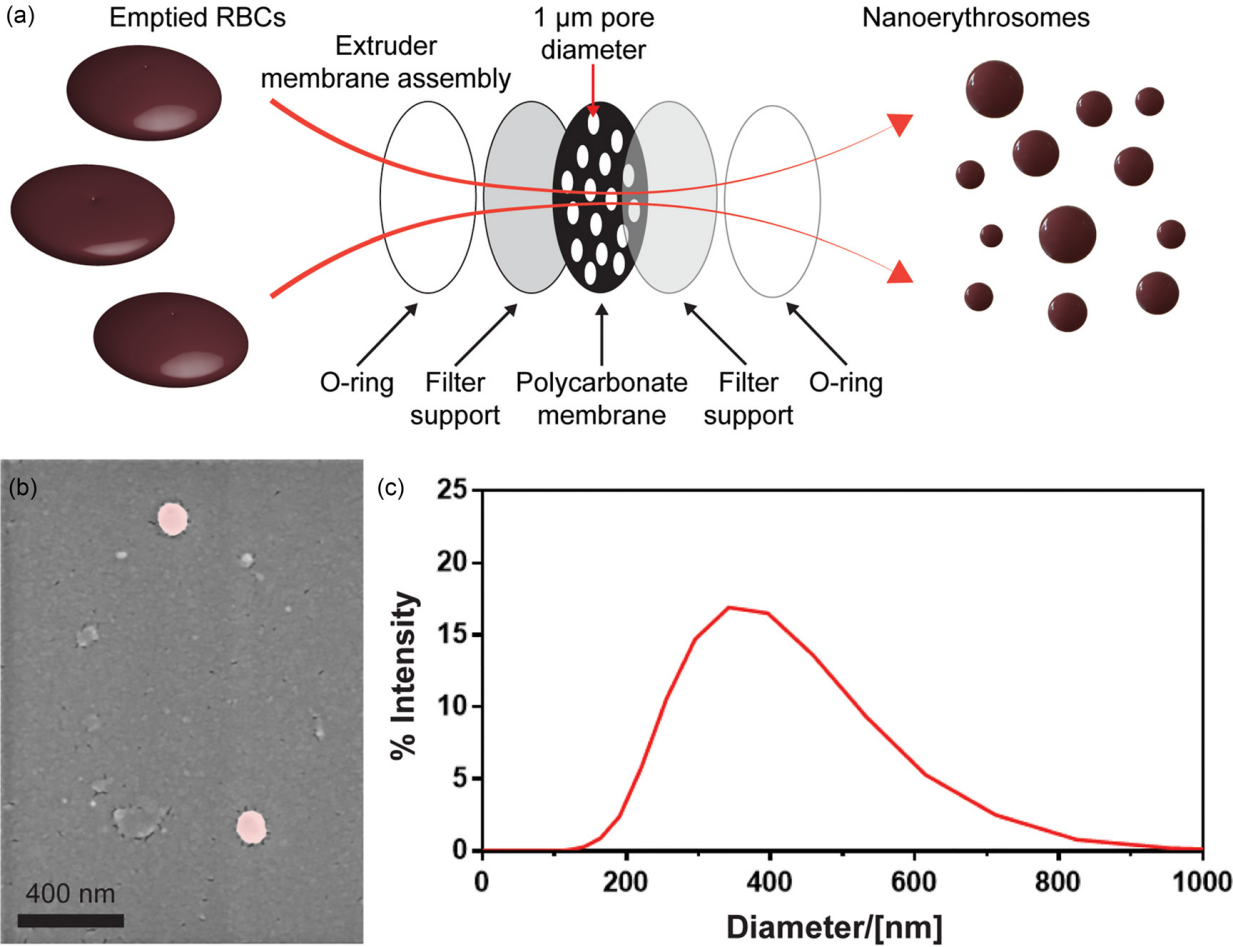
Fabrication and physical characterization of the nanoerythrosomes. (a) Nanoerythrosomes are fabricated by extrusion of the emptied RBCs, also known as RBC ghosts, through a track-etched membrane with 1 *μ*m pores. (b) SEM image and (c) DLS measurement of the fabricated nanoerythrosomes.

The nanoerythrosomes were fabricated by following three consecutive processes. Initially, RBCs were isolated from murine blood by using the Percoll gradient assay. Three specific layers were obtained inside the Percoll density gradient, following the centrifugation of the whole blood. While the top and bottom layers mainly contained leucocytes and cell debris, respectively, RBCs were concentrated in the middle layer as a dark red ring and slowly collected into a new tube without disturbing the top and bottom layers. In the second process, the cytoplasmic content of the RBCs was emptied by using a hypotonic-isotonic treatment procedure, which led to the formation of approximately 100 nm pores on the cell membranes of the RBCs,^44^ which allowed diffusion of the cytoplasmic content of the cells into the solution. RBC membranes were resealed after treating with the isotonic solution to preserve the membrane integrity prior to cell extrusion. In the final process, the emptied RBCs were slowly passed through a polycarbonate membrane with a pore size of 1 *μ*m using a cell extruder [Fig. 2(a)]. The membrane pore size and the extrusion speed determine the size and the uniformity of the fabricated nanoerythrosomes.^45^ SEM analyses of the fabricated nanoerythrosomes showed that the cell membranes were properly re-folded and formed hollow nanovesicles [Fig. 2(b)]. The morphology of the fabricated nanoerythrosomes was similar to the previously reported results in the literature.^34,46^ Furthermore, DLS investigations showed a polydisperse population ranging from approximately 100 nm to 1 *μ*m diameter with a maximum peak around 350 nm [Fig. 2(c)]. The polydisperse nanoerythrosome population with vesicle sizes smaller than the extruded pore size was also in accordance with the literature.^34,41^

**FIG. 3.**
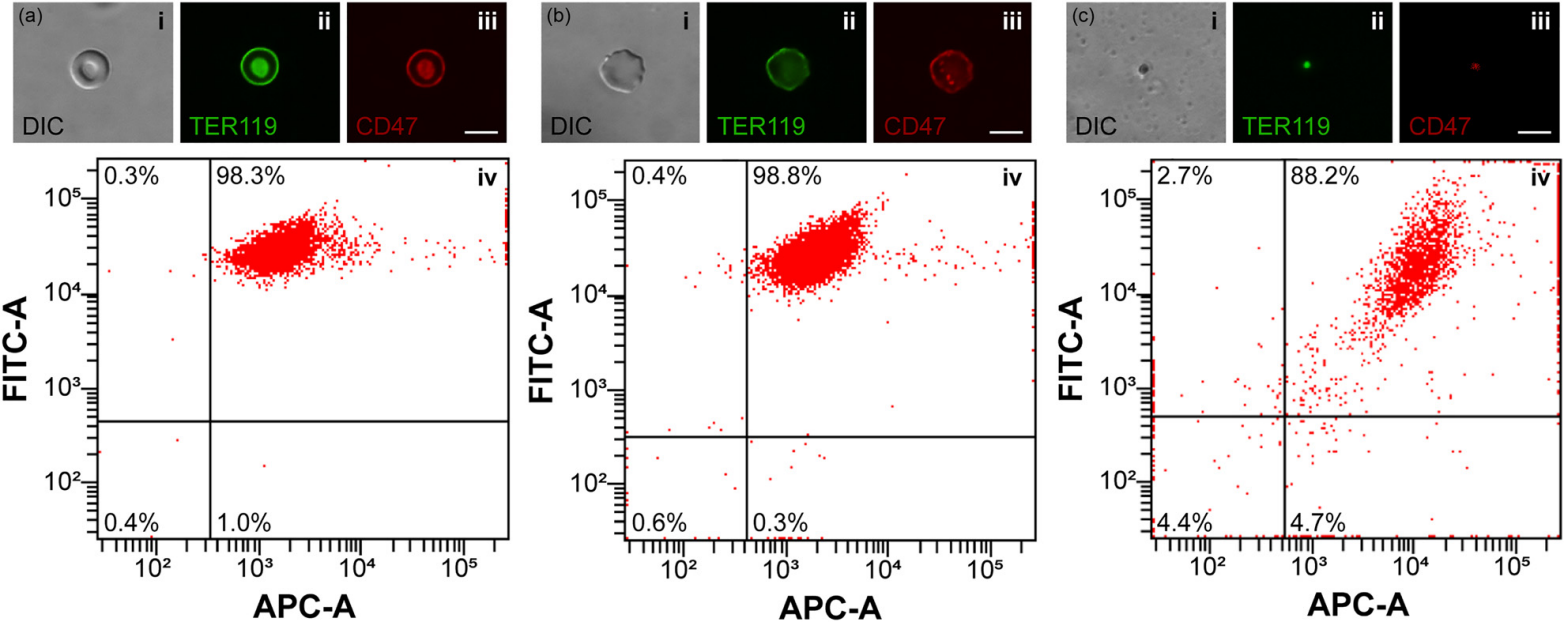
Analyses of the important membrane proteins on the RBCs, the emptied RBCs, and the nanoerythrosomes. (a)–(c) Preservation of the essential membrane proteins, TER119 and CD47, on the RBCs (a), and the emptied RBCs (b), and the nanoerythrosomes (c) were analyzed via inverted fluorescence microscopy (top row) and flow cytometry (bottom row) techniques. More than 88% of all nanoerythrosomes showed double-positive results for TER119 and CD47 proteins on their outer membranes. Scale bars are 5 *μ*m.

Following the fabrication and the morphological characterization of the nanoerythrosomes, it is crucial to determine the preservation of essential cell membrane proteins. The presence of TER119 and CD47 proteins on the fabricated nanoerythrosomes was investigated using fluorescence microscopy and flow cytometry analyses after each sequential process: separation of the RBCs, hypotonic-isotonic treatment for preparation of the emptied RBCs, and extrusion of the nanoerythrosomes fabrication. After the Percoll gradient assay, the isolated RBCs were stained with green fluorescent-labeled TER119 antibodies and red fluorescent-labeled CD47 antibodies. The fluorescence microscopy investigation revealed that the isolated RBCs displayed TER119 and CD47 proteins on their cell membrane [Figs. 3(a-i)–3(a-iii)]. The flow cytometry investigation of the isolated RBCs presented that 98.3% of the population was double positive for both TER119 and CD47 proteins [Figs. 3(a-iv) and S3(a)]. After the hypotonic-isotonic treatment process, the emptied RBCs still had TER119 and CD47 proteins on their membranes and gave fluorescent signals upon treatment with green fluorescent labeled TER119 antibodies and red fluorescent-labeled CD47 antibodies [Figs. 3(b-i)–3(b-iii)]. The investigation of the emptied RBCs with flow cytometry analyses further showed that 98.8% of the population was double positive for both TER119 and CD47 proteins [Figs. 3(b-iv) and S3(b)]. Only 0.6% of the emptied RBC population was simultaneously negative for both TER119 and CD47 proteins indicating that the hypotonic-isotonic treatment process also did not significantly affect the RBCs. The staining of the fabricated nanoerythrosomes with green fluorescent labeled TER119 antibodies and red fluorescent-labeled CD47 antibodies showed the presence of these proteins on the membrane of the fabricated nanoerythrosomes [Figs. 3(c-i)–3(c-iii)]. Moreover, the flow cytometry results revealed that 88.2% of the fabricated nanoerythrosome population was double positive for both TER119 and CD47 proteins, whereas 4.4% of the population was negative for both TER119 and CD47 proteins on their membranes [Figs. 3(c-iv) and S3(c)]. Despite the increased double-negative population percentage, due to the extrusion process, still a significant percentage of the fabricated nanoerythrosomes had TER119 and CD47 proteins (Table S1), which were essential for the fabrication of the biohybrid microswimmers and their future medical applications.

**FIG. 4.**
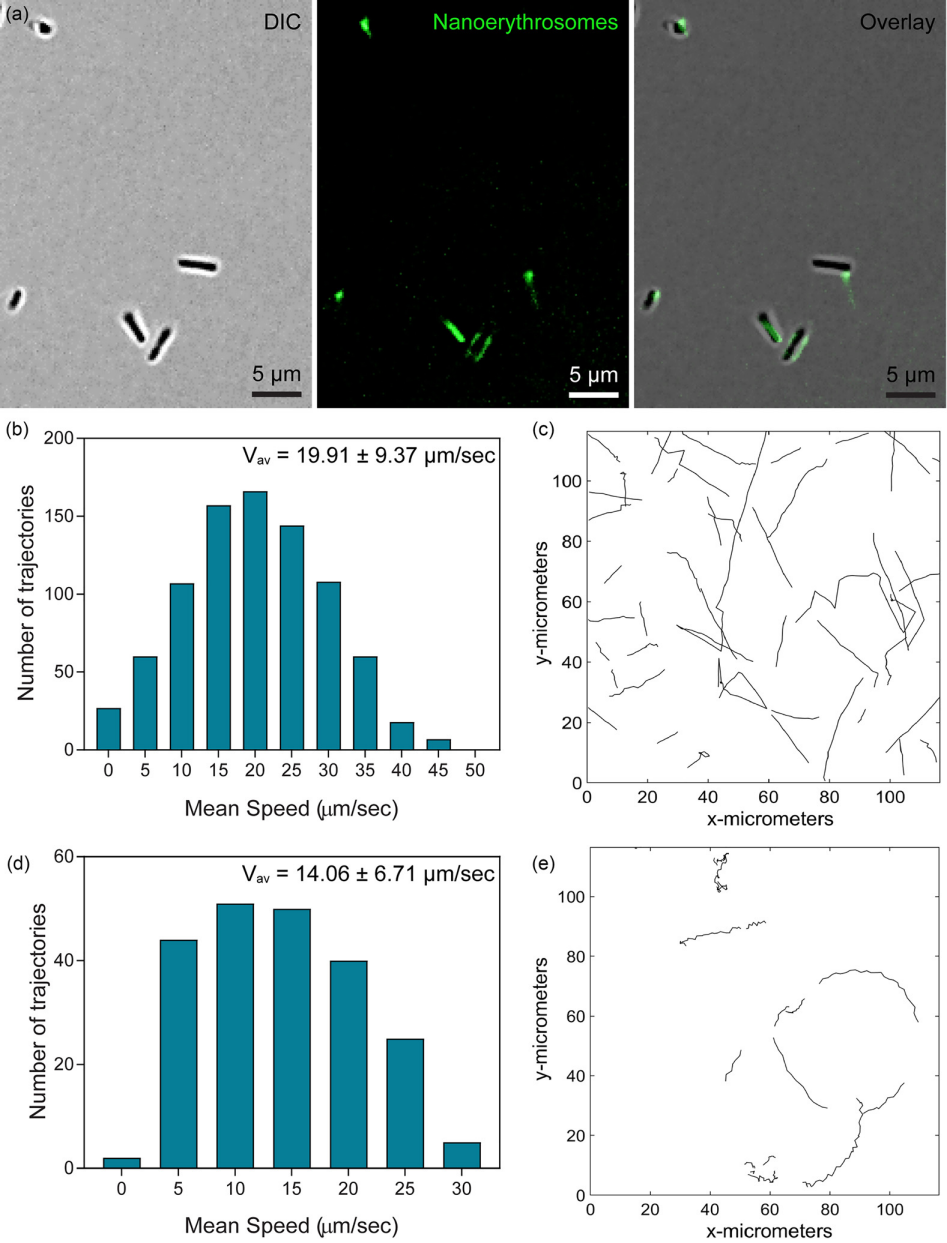
Swimming performance of the biohybrid bacterial microswimmers compared to the free-swimming genetically engineered bacteria. (a) Conjugation of the nanoerythrosomes to genetically engineered bacteria species to fabricate biohybrid microswimmers. Nanoerythrosomes were stained with biotin-conjugated AlexaFluor 488 fluorophores for the fluorescence microscopy analysis. 2D motility characterization of the free-swimming genetically engineered bacteria [(b) and (c)] and the biohybrid bacterial microswimmers [(d) and (e)] showed a mean speed of 19.91 ± 9.37 *μ*m/s and 14.06 ± 6.71 *μ*m/s, respectively.

Conjugation of the biotin-displaying *E. coli* MG1655 substrain with the nanoerythrosomes labeled with biotinylated TER119 was achieved by streptavidin coupling. Nanoerythrosome conjugation to the bacteria was confirmed using fluorescence microscopy analyses [Fig. 4(a)]. After conjugation of the biotinylated nanoerythrosomes to the biotin-displaying genetically engineered *E. coli* MG165 substrain via streptavidin coupling proteins, motility performance of the resulting biohybrid microswimmers was analyzed in sealed microchannels (supplementary material, Movie S1). The mean swimming speed of the free bacteria was 19.91 ± 9.37 *μ*m/s and displayed linear swimming trajectories [Figs. 4(b) and 4(c)]. The nanoerythrosome-functionalized biohybrid microswimmers had 14.06 ± 6.71 *μ*m/s average swimming velocity and demonstrated wavy trajectories, which may be due to the presence of the artificial carriers on their membranes [Figs. 4(d) and 4(e)]. In addition, it was observed that the interaction of the nanoerythrosomes with bacterial flagella did not completely terminate the propulsion of the biohybrid microswimmers. Microswimmers with nanoerythrosomes attached to the bacterial flagella demonstrated the helical shape of the rotating bacterial flagella while swimming inside the sealed microchannels (supplementary material, Movie S2). Finally, the average velocity of the biohybrid microswimmers presented here was greater than the previously reported swimming speed performance of other biohybrid designs driven by *E. coli* strains.^5,13^ Biohybrid microswimmers powered by microorganisms, including bacteria and microalgae, operate at a low Reynolds number. In the low Reynolds number flow regime, fluid motion is governed by the Stokes equation and inertial effects on the microswimmer body are negligible. Therefore, fluid drag force ($F_{d}$) for the biohybrid microswimmers can be expressed as^8,47^

$$F_{d}=-6\pi \eta \left(R_{A}+R_{c}\right)v,$$ (1)

where η is the dynamic viscosity of the fluid medium, $R_{A}$ and $R_{c}$ are radius of the bioactuator and the integrated cargo carrier, respectively, and *v* is the velocity of the biohybrid microswimmer. Higher swimming performance of the biohybrid microswimmers shown here could be ascribed to the relatively small size of the utilized synthetic units, which results in a much smaller viscous drag on the biohybrid microswimmer body. For instance, fluidic drag forces acting on an *E. coli*-powered biohybrid microswimmer with a 4 *μ*m cargo carrier and operating inside a physiological buffer, e.g*.,* phosphate buffered saline (PBS), are approximately three times larger than those on a biohybrid microswimmer with a 200 nm cargo carrier operating in similar conditions. Such a decrease in fluidic drag forces acting on the biohybrid microswimmer could enhance their operation in complex biological environments with higher viscosities and smaller pore sizes.

## DISCUSSION

Construction of active cargo delivery systems using microorganisms and biological materials, which can be obtained from the human body, presents promising potential to revolutionize various medical operations, including drug delivery and cancer treatment, in hard-to-reach body locations. Selection of bioactuators naturally found in the human body, such as *E. coli*, and cargo carriers, such as RBCs, which could be easily obtained from patients, is crucial for the development of next-generation biohybrid microswimmers with superior medical characteristics, such as lower immunogenicity, decreased protein corona formation, enhanced circulation time, and navigation inside complex body environments.^1,38,39,48^

Biological actuators employed in biohybrid microswimmers should be selected for the desired medical applications. While some bacteria species [e.g., magnetotactic bacteria (MTB)] possess high motility and inherent magnetic steering capability, their survival in bodily conditions ia limited to an hour and therefore would be suited best for short-term rapid interventions. On the other hand, other bacteria species (e.g., *E. coli*), although slower in speed, are evolutionarily optimized to survive and function in bodily conditions and can be used for long-term medical operations.^49,50^ For the cargo carrier selection, one of the crucial parameters is the size of the cargo carrier, which directly affects the propulsion performance and navigation in confined spaces. Therefore, the use of nano-sized carriers in biohybrid microswimmer design would be advantageous for fast propulsion and motility in complex biological environments. While previous biohybrid microswimmer designs have incorporated nanoliposomes and nanoscale polymeric particles as cargo carriers,^16,51,52^ the nanocarriers used in our design, nanoerythrosomes, are naturally driven and, therefore, contain important membrane proteins that are crucial to avoid unwanted interactions with the immune cells. Furthermore, the presence of well-known and studied membrane proteins would also allow facile modification of the cargo carrier surface and addition of versatile functionalities, such as targeting.

While preparing the nanoerythrosomes, it is important to preserve cell membrane proteins of the RBCs to exploit their physical and chemical functions to the best extent. In this study, preservations of two important cell membrane elements, TER119 and CD47, were examined. TER119, which is a 52 kDa protein and the linage marker of the mature RBCs,^43^ was utilized to non-invasively conjugate the nanoerythrosome to the genetically engineered bacteria through biotin–streptavidin interaction, and therefore, its integrity and preservation were crucial for the overall success of the biohybrid design. CD47, a 50 kDa membrane protein which serves as a marker of “self-protein” and prevents macrophage uptake through its interaction with the inhibitory signal regulatory protein alpha,^34^ was analyzed to obtain information about the possible future applications of the proposed biohybrid microswimmer, including enhanced circulation time and partial immune evasiveness. In addition, CD47 inhibits phagocytic uptake by counteracting opsonization based pro-phagocytic signals,^53^ and it would have paramount importance to enhance the operation window of biohybrid microswimmers via postponing their immune clearance in the future medical applications.

Biotin–streptavidin interaction, used in this work, is one of the strongest non-covalent interactions found in nature. Streptavidin has four identical subunits that can individually interact with four different biotin molecules.^13,54^ Streptavidin is also resistant to extreme pH conditions and has high thermal stability. The use of a recombinant biotin-displaying *E. coli* MG1655 substrain enables non-invasive fabrication of biohybrid microswimmers with preserved flagella composition and efficient propulsion compared to earlier reports utilizing harsh chemical reactions for conjugation.^11^ Furthermore, smaller size and density of nanoerythrosomes, compared to micrometer-scale polymeric or metallic carriers used in other reports, enables the higher average speed of the biohybrid microswimmers described here.^55^ Additionally, utilization of nanoerythrosomes, rather than emptied RBCs, as cargo carriers could ease and enhance the navigation of biohybrid microswimmers inside complex bodily environments due to the decreased fluidic drag force governed on the microswimmers, and also could lead to locomotion of the microswimmers inside extracellular spaces that would be as small as bacterial dimensions. Future studies will focus on cargo carrying and penetration capabilities of the nanoerythrosome-functionalized biohybrid microswimmers through the biological barriers and into the tumor microenvironment. Overall, in this work, the establishment of a nanoerythrosome-functionalized biohybrid microswimmer design has been demonstrated and could be employed in future applications of biohybrid microswimmers in bacteria-mediated immunotherapy and in personalized medical operations.

## METHODS

### Bacteria culture

A genetically engineered *E. coli* MG1655 substrain was initially cultured overnight from single colony at 37 °C and 200 rpm in tryptone broth (TB) medium [10 g tryptone (Sigma-Aldrich^®^, St. Louis, MO) and 5 g NaCl (Sigma-Aldrich, St. Louis, MO) in 1 l ddH_2_O, pH 7] containing 50 *μ*g/ml kanamycin (KM) (Sigma-Aldrich, St. Louis, MO) and 100 *μ*g/ml ampicillin (AMP) (Sigma-Aldrich, St. Louis, MO). Then, 100 *μ*l of the overnight bacterial culture was transferred into fresh 10 ml TB medium which consisted of 1 *μ*M biotin (Sigma-Aldrich, St. Louis, MO), 50 *μ*g/ml KM, and 100 *μ*g/ml AMP and further cultured at 34 °C and 270 rpm for 2h. Subsequently, 100 *μ*M isopropyl β-D-1-thiogalactopyranoside (IPTG) (Sigma-Aldrich, St. Louis, MO) was added into the medium and the bacteria were further cultured at 34 °C and 270 rpm until their optical density at 600 nm (OD_600_) reached 0.6, which was measured with a Synergy HTX multi-mode plate reader (BioTek Instruments, Winooski, VT).

### Isolation of the RBCs with Percoll assay

Murine whole blood was obtained from healthy C57BL/6 mice, which were kindly provided by the animal welfare office of the Eberhard Karls University and the University Hospital Tübingen. Ethics approval is not required for the obtained murine whole blood samples. RBCs were isolated and purified from the obtained murine whole blood using Percoll assay. Briefly, the Percoll density gradient was prepared inside a 15 ml centrifuge tube using three different Percoll solutions, which were 21% (v/v), 38% (v/v), and 80% (v/v) Percoll (GE Healthcare Biosciences AB, Uppsala, Sweden) in 1× PBS (Thermo Fisher Scientific, Waltham, MA). Initially, 5 ml 80% (v/v) Percoll solution was put into the centrifuge tube and then sequentially 3 ml 38% (v/v) Percoll solution and 2 ml 21% (v/v) Percoll solution were carefully transferred on top of it. Then, 500 *μ*l murine whole blood was diluted with 500 *μ*l 1× PBS and the diluted whole blood was gently transferred into the centrifuge tube without disturbing the Percoll solutions. Finally, the prepared sample was centrifuged at 4 °C and 800*g* for 20 min, and following the centrifugation procedure the formed dark red middle phase, containing the RBCs, was carefully transferred into a new tube for further uses.

### Preparation of the nanoerythrosomes

Before the fabrication of the nanoerythrosomes, cytoplasmic contents of the RBCs were emptied as previously described.^13^ Initially, RBCs were washed two times with 10 ml 1× PBS and then incubated in a hypotonic solution [1× PBS:ddH_2_O (1:1)] at 4 °C and 1200 rpm for 15 min. After that, the sample was centrifuged 4 °C and 750*g* for 5 min and the pellet, containing the emptied RBCs, was resuspended in 1 ml 1× PBS. Next, the sample was further incubated at 37 °C and 1200 rpm for 15 min to properly reseal the emptied RBCs. Finally, the emptied RBCs were centrifuged at RT and 750*g* for 5 min and dissolved in 1 ml 1× PBS to utilize in the nanoerythrosome fabrication process. Nanoerythrosomes were fabricated from the emptied RBCs using an extruder (Avanti Polar Lipids, Alabaster, AL). Briefly, 1 ml emptied RBCs were gently pushed through a polycarbonate membrane filter, which had 1 *μ*m diameter pore sizes and was placed into the extruder, for eight cycles at 35 °C, and then washed with 1× PBS for two times via centrifugation at RT and 30 000*g*. Finally, the nanoerythrosomes were dissolved in 1 ml 1× PBS and stored at 4 °C for further uses.

### Characterization of the nanoerythrosomes

The prepared nanoerythrosomes were initially characterized by using dynamic light scattering (DLS) and scanning electron microscopy (SEM) techniques. DLS analysis was performed with a Zetasizer Nano ZS (Malvern Instruments, Worcestershire, UK). For the measurement, the nanoerythrosomes were diluted 1:100 in 1× PBS and their sizes were measured successively five times. SEM analysis was performed with a Zeiss Gemini 550 scanning electron microscope (Carl Zeiss, Oberkochen, Germany) with an accelerating voltage of 3 keV and an in-lens detector. Before the SEM analysis, nanoerythrosomes were fixed on a track-etched polycarbonate membrane filter (Whatman, Maidstone, UK) that had 100 nm diameter pore sizes. Briefly, the sample was incubated in 2.5% (v/v) glutaraldehyde solution (Sigma-Aldrich, St. Louis, MO) at 4 °C for 15 min and then washed three times with 1× PBS. Next, the samples were dehydrated via sequentially incubating in ethanol solutions with increasing concentrations of 20% (v/v), 40% (v/v), 60% (v/v), and 80% (v/v) and absolute ethanol for 3 min. After that, the samples were dried using an automated Leica EM CPD300 critical point dryer (Leica Microsystems, Wetzlar, Germany), and finally, the dried samples were coated with 10 nm gold using a Leica EM ACE600 sputter coater (Leica Microsystems, Wetzlar, Germany).

### Investigation of the nanoerythrosomes with confocal microscopy and flow cytometry

Various antibodies with different fluorescent tags, including AlexaFluor^®^ 488 (BioLegend, San Diego, CA), AlexaFluor 647 (BioLegend, San Diego, CA) and fluorescein isothiocyanate (FITC) (BioLegend, San Diego, CA), were used to investigate the presence of desired cell membrane proteins, including CD47 and TER119, on the prepared nanoerythrosomes. Confocal laser microscopy and flow cytometry techniques were used for qualitative and quantitative analysis of the desired cell membrane proteins. For the flow cytometry investigations, 90 *μ*l nanoerythrosomes were stained with 10 *μ*l fluorescent antibodies, including AlexaFluor 488 anti-mouse TER-119/erythroid cells (BioLegend, San Diego, CA) and AlexaFluor 647 anti-mouse CD47 (BioLegend, San Diego, CA), in fluorescence-activated cell sorting (FACS) buffer [0.2% (w/v) bovine serum albumin (BSA), and in 1× PBS (pH 7.4)] (BD Biosciences, East Rutherford, NJ). Initially, nanoerythrosomes were incubated with the antibodies at 4 °C in the dark for 0.5 h. Then, the sample was washed two times at RT and 30 000*g* and dissolved in 100 *μ*l 1× PBS to utilize in the flow cytometry measurements. During the measurements, at least 10 000 events were acquired using a LSR Fortessa flow cytometer (BD Biosciences, East Rutherford, NJ) and results were presented with DIVA software (BD Biosciences, East Rutherford, NJ).

### Fabrication and characterization of the biohybrid microswimmers

Biohybrid microswimmers were fabricated through biotin-streptavidin interaction by using the prepared nanoerythrosomes and a genetically engineered *E. coli* MG1655 substrain. The genetically engineered bacteria strain contained a pOS233 plasmid, which was modified in the Ag43 region to express the FLAG epitope and biotin attachment peptide. Briefly, after the bacteria culture reached 0.6 OD_600_, 1 ml bacteria culture was rinsed two times with motility buffer [10 mM K_2_HPO_4_, 10 mM KHPO_4_, 67 mM NaCl, 0.1 mM EDTA and 1% (w/v) glucose, pH 7] at RT and 2000*g* and then incubated in motility buffer that contained 100 *μ*g/ml streptavidin (Sigma-Aldrich, St. Louis, MO) at 37 °C and 200 rpm for 1 h. After the incubation period, the sample was rinsed two times at RT and 2000*g* to remove the unbounded streptavidin molecules and then resuspended in 1 ml motility buffer to use in the conjugation procedure. In parallel, nanoerythrosomes were modified with biotin-conjugated anti-TER119 antibodies at 37 °C for 1 h and then washed two times with 1× PBS at RT and 30 000*g*. After that streptavidin-functionalized bacteria were mixed with biotin-modified nanoerythrosomes with 1:20 ratio and incubated at 35 °C and 1200 rpm for 2 h to realize the fabrication of the biohybrid microswimmers. For the confocal laser microscopy analysis, biohybrid microswimmers were further stained with AlexaFluor 488 anti-TER119 and AlexaFluor 647 anti-CD47 antibodies. For the motility analysis, biohybrid microswimmers were injected into microchannels (75 *μ*m height × 2 mm width × 10 mm length), composed of laser-cut poly(methyl methacrylate) pieces and double-sided adhesive films attached to cover glasses, and examined using an inverted fluorescent microscope with a 60× oil-immersion objective.

For the motility analysis, biohybrid bacterial microswimmers were tracked from over 20 video recordings (28 frames per second) obtained using a Nikon Instruments, Inc. Eclipse Ti-E spinning disk confocal microscope with a 60× magnification immersion oil objective. Mean velocity and trajectories of biohybrid bacterial microswimmers and free-swimming genetically engineered *E. coli* MG1655 substrain were computationally analyzed using an in-house 2D object tracking software developed in MATLAB (MathWorks, Natick, MA).

## AUTHOR'S CONTRIBUTIONS

N.B., O.Y., and Y.A. contributed equally to this work.

## SUPPLEMENTARY MATERIAL

See the supplementary material for the supplementary figures and movie of the nanoerythrosome-functionalized biohybrid microswimmers.
